# Sensitivity of leukemic T-cell lines to arsenic trioxide cytotoxicity is dependent on the induction of phosphatase B220/CD45R expression at the cell surface

**DOI:** 10.1186/1476-4598-13-251

**Published:** 2014-11-19

**Authors:** Mohcine Benbijja, Amine Mellouk, Pierre Bobé

**Affiliations:** Institut Jacques Monod, CNRS, Université Paris Diderot, Paris, France; INSERM U1012, Université Paris-Sud, Le Kremlin Bicêtre, Orsay, France; INSERM U757, Université Paris-Sud, Orsay, France

**Keywords:** As_2_O_3_-based therapy, Leukemia, T lymphocyte, Membrane tyrosine-phosphatase B220/CD45R, Membrane-bound HSP70, Fas/Fas ligand pathway, Cell death, Caspase activation, NF-κB p50

## Abstract

**Background:**

Arsenic trioxide (As_2_O_3_) is highly effective in treating acute promyelocytic leukemia (APL), but shows more variable therapeutic efficacy for other types of hematological malignancies. Previously, we reported that As_2_O_3_ selectively eliminates pathogenic B220-expressing T cells in autoimmune MRL/*lpr* mice. We investigated herein the relationship between As_2_O_3_ sensitivity of leukemic T-cell lines and the expression levels of the B220 isoform of transmembrane tyrosine phosphatase CD45.

**Methods:**

GSH content, O2^-^ production, and B220, HSP70, Fas and FasL membrane expression was measured by flow cytometry. Subcellular localization of B220 was determined by imaging flow cytometry. Cell death was analyzed by morphological changes, annexin V and propidium iodide staining, and caspase 8 and 9 activation. B220 mRNA expression was analyzed by RT-PCR. Activated NF-κB p50 was quantified by a DNA binding ELISA.

**Results:**

We selected human (Jurkat, Jurkat variant J45.01, HPB-ALL) and mouse (EL-4, BW5147, L1210) T-cell lines for their marked differences in As_2_O_3_ sensitivity over a large range of doses (1 to 20 μM). Differences in redox status cannot explain the dramatic differences in As_2_O_3_ sensitivity observed among the T-cell lines. Unexpectedly, we found that B220 is differentially induced on As_2_O_3_-treated T-cell lines. As_2_O_3_ treatment for 24 h induced low (HPB-ALL), intermediate (Jurkat) and high (EL-4, BW5147) levels of B220 membrane expression, membrane-bound HSP70 and cell death, but inhibited NF-κB p50 nuclear translocation. When high levels of B220 expression were achieved with low doses of As_2_O_3_, the T-cell lines died by apoptosis only. When high doses of As_2_O_3_ were required to induce B220 expression, leukemic T cells died by both apoptosis and necrosis.

**Conclusions:**

Cellular redox status is not essential for As_2_O_3_ sensitivity of leukemic T cells, suggesting the existence of additional factors determining their sensitivity to As_2_O_3_ cytotoxicity. Phosphatase B220 could be such a factor of sensitivity. As_2_O_3_ treatment inhibits NF-κB p50 nuclear translocation, and induces B220 expression and cell death in a dose and time dependent manner. The levels of B220 induction on leukemic T cells strictly correlate with both the extent and form of cell death, B220 might therefore play a checkpoint role in death pathways.

**Electronic supplementary material:**

The online version of this article (doi:10.1186/1476-4598-13-251) contains supplementary material, which is available to authorized users.

## Background

Arsenic trioxide (As_2_O_3_) shows impressive efficacy in the treatment of patients with acute promyelocytic leukemia (APL) [1–4]. As_2_O_3_ induces clinical remission in APL patients by multiple mechanisms [5]. As_2_O_3_ promotes cell differentiation at low concentrations [6, 7], whereas it induces apoptosis at higher concentrations [8]. The high sensitivity of the APL cell line NB4 to As_2_O_3_-induced cytotoxicity is associated to its low content of reduced glutathione (GSH) and increased production of reactive oxygen species (ROS) [9–11]. Although most studies have been focused on the APL, As_2_O_3_ could be beneficial against various hematopoietic malignancies and solid tumors [12]. Moreover, we have shown that As_2_O_3_ also possesses immunomodulatory properties, and might be a therapeutic agent for autoimmune diseases. Indeed, As_2_O_3_ selectively eliminates the pathogenic B220-expressing double negative (DN) CD4^-^CD8^-^ T cells that accumulate in autoimmune MRL/*lpr* mice due to the *lpr* mutation of the death receptor Fas [13, 14].

In normal murine and human T cells, CD4^+^ and CD8^+^ effector T cells massively induce the expression of transmembrane tyrosine phosphatase B220 before undergoing apoptosis by the Fas/Fas ligand (FasL) pathway [15, 16]. In Fas-deficient mice and patients, CD4^+^ and CD8^+^ effector T cells also express the B220 molecules at their surface, but then they downregulate their CD4 or CD8 molecules while maintaining B220 plasma membrane expression. B220 (or CD45RABC) is one of the five isoforms of the transmembrane tyrosine phosphatase CD45 found on lymphocytes. CD45 isoforms are generated by cell-type and activation-state specific alternative splicing of exons 4/A, 5/B, and 6/C encoding domains at the NH_2_-terminus. Naive T cells express high molecular weight CD45 isoforms (CD45RA or CD45RB) containing the A domain in humans or the B domain in mice whereas effector/memory T cells expressed the low molecular weight isoform CD45RO lacking extracellular domains A, B and C. All CD45 isoforms share the same intracellular region, which contains two phosphatase domains. Although the function of each isoform remains unknown, it is well established that CD45 phosphatase activity is crucial for lymphocyte development, and antigen and cytokine receptor signaling [17–19]. CD45 might also regulate apoptosis of T and B lymphocytes [20–22].

In this study, we found that murine (EL-4, BW5147, L1210) and human (Jurkat, CD45-deficient Jurkat variant, HPB-ALL) leukemic T-cell lines dramatically differed in their sensitivity to As_2_O_3_-induced cell death. In contrast with previous findings in APL cell line NB4 [9, 10], these differences in As_2_O_3_ sensitivity are independent of intracellular GSH content and O_2_^-^ production. Unexpectedly, we found that As_2_O_3_ differently induced B220 cell surface expression in the leukemic T-cell lines in a dose- and time-dependent manner. Moreover, the levels of B220 expression correlated with the sensitivity of these T-cell lines to As_2_O_3_. Induction of B220 membrane expression by As_2_O_3_ treatment is reminiscent of that observed on antigen-activated normal T-cell blasts before undergoing apoptosis [15, 16]. Therefore, the leukemic T-cell lines were activated with calcium ionophore A23187, which triggers both cell activation and cell death. Calcium ionophore A23187 also induced B220 expression and cell death, but with reverse efficiencies in the leukemic T-cell lines compared to As_2_O_3_. In addition, T-cell lines treated with A23187 most probably died by an activation-induced cell death mechanism since the T-cell activation marker CD69 is expressed before B220 expression and cell death. In contrast, CD69 was not detected on As_2_O_3_-treated cells, indicating that B220 expression occurs independently of leukemic T-cell activation. Surprisingly, we found that B220 is expressed constitutively on L1210 T cells. L1210 cells were highly sensitive to A23187 treatment whereas they were highly resistant to As_2_O_3_ cytotoxicity, indicating that the constitutive high-level expression of B220 did not favor cell death triggered by As_2_O_3_. B220 induction on the T-cell lines after treatment with As_2_O_3_ or calcium ionophore A23187 strictly correlates with sensitivity to cell death, emphasizing the role of B220 as a proapoptotic factor. However, our data indicate that As_2_O_3_ and A23187 trigger B220 induction and cell death through different upstream signaling pathways. Different signaling pathways, such as the c-Jun NH_2_-terminal kinase, have been implicated as mediators of the cytotoxic effects of As_2_O_3_ in the APL cell line NB4 [23]. Here, we show that high induction of B220 expression on leukemic T cells is a determining factor leading to As_2_O_3_-triggered cell death. Thus we hypothesize that B220 might play a checkpoint role in death pathways.

## Results

### Human and mouse leukemic T-cell lines exhibit different sensitivities to As_2_O_3_-induced growth inhibition and cytotoxicity

**Figure 1:**
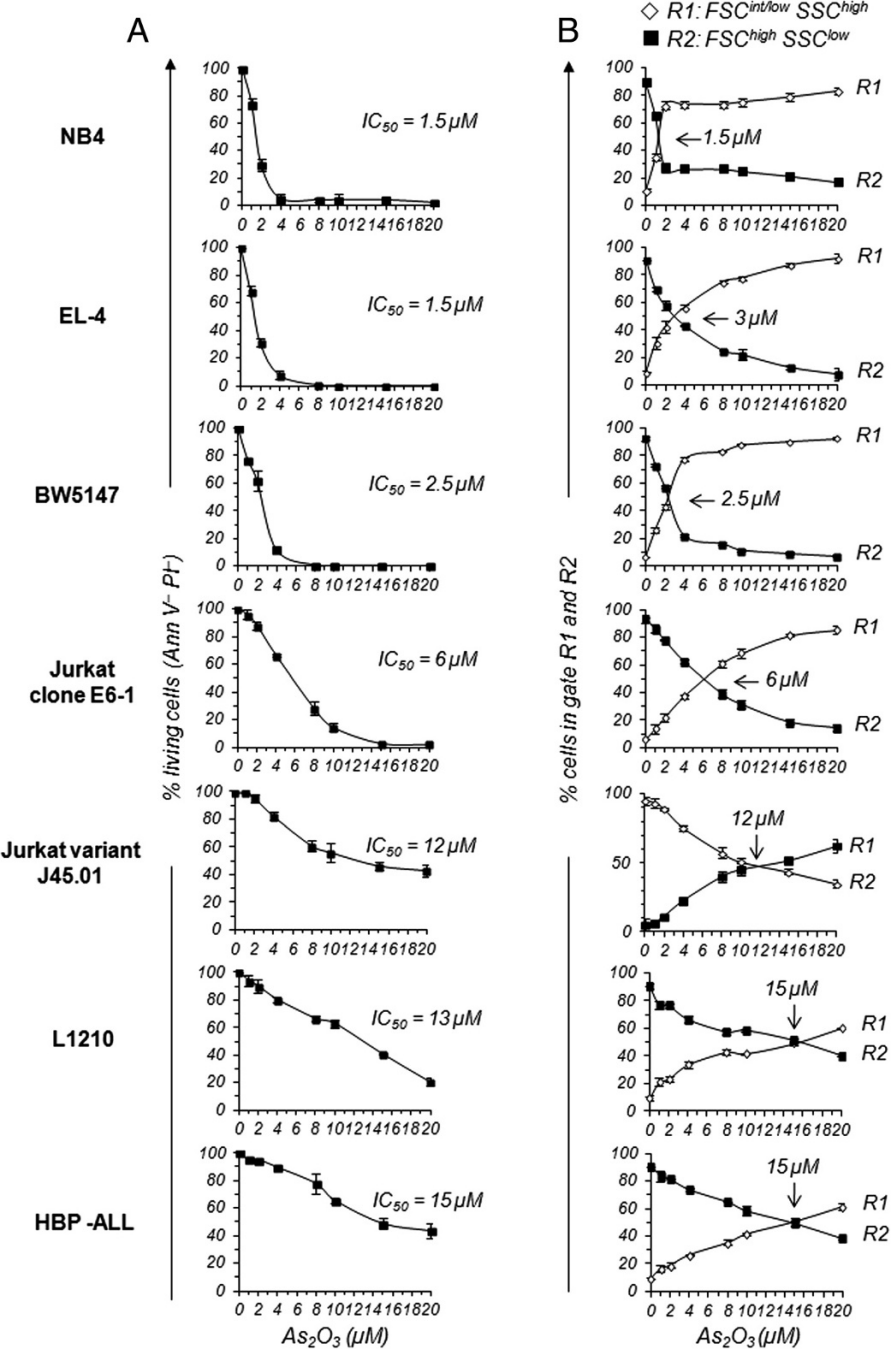
**Viability and cell morphology of various T-cell lines cells upon As**_**2**_ **O**_**3**_ **-treatment. (A)** APL-derived NB4 cells as well EL-4, BW5147, L1210, Jurkat (clone E6-1), CD45-deficient Jurkat variant (J45.01) and HPB-ALL T-cell lines were treated with As_2_O_3_ for 24 h with doses ranging from 1 to 20 μM. T-cell lines were then stained with Annexin V and PI, and the percentages of living cells (Ann V^-^ PI^-^) determined by flow cytometry. At least 20,000 events were analyzed for each sample. Graphs show mean percentages ± SE of Ann V^-^ PI^-^ cells from more than 4 independent experiments. IC_50_ in the graphs indicates the concentration of As_2_O_3_ that kills 50% of the cells. **(B)** Cells were analyzed by flow cytometry with respect to size (FSC) and granulosity (SSC). Regions R1 and R2 identified on a FSC vs. SSC dot plot encompassed cells with FSC^intermediate(int)/low^SSC^high^ and FSC^high^SSC^low^, respectively. At least 20,000 events were analyzed for each sample. Graphs show mean percentages ± SE of cells located in region R1 (◊) and R2 (■) at the indicated concentration of As_2_O_3_. Numbers in the graphs indicate the concentration of As_2_O_3_ at which 50% of the cells shifted from region R2 to region R1. Four independent experiments were performed for NB4 and J45.01 cells, and more than 10 for EL-4, BW5147, L1210, Jurkat and HPB-ALL cells.

In addition to APL-derived NB4 cells, As_2_O_3_ kills various cancer cell types [12]. However, As_2_O_3_ sensitivity varies considerably among tumor cell lines. In the present study, we determined the sensitivity to As_2_O_3_-induced cytotoxicity and growth inhibition of human (Jurkat and HPB-ALL) and murine (EL-4, BW5147 and L1210) leukemic T-cell lines. The five T-cell lines along with APL-derived NB-4, known for its high sensitivity to As_2_O_3_ cytotoxicity, were treated with As_2_O_3_ in doses ranging from 1 to 20 μM for 12, 24 and 48 h. Then, the percentage of living cells was determined by Annexin V and propidium iodide (PI) staining, and flow cytometry. We rapidly abandoned treatments for 12 h and 48 h. Indeed, 100% EL-4 and BW5147 cells were killed after 24 h of treatment with low doses of As_2_O_3_, whereas HPB-ALL and L1210 cells showed no sensitivity to As_2_O_3_ cytotoxicity before 24 h of treatment, even with high doses (data not shown). Because 24 h of treatment was the optimal time to observe significant differences in As_2_O_3_ cytotoxicity among T-cell lines, this duration was used in subsequent experiments. Sensitivity to As_2_O_3_ varied considerably among the T-cell lines (Figure 1A). While around 3% NB4, 6% EL-4 and 13% BW5147 cells remained alive (Annexin V^-^ and PI^-^) at a concentration of 4 μM As_2_O_3_, remarkably, the percentages of living cells were around 65% for Jurkat cells, and between 80% and 90% for HPB-ALL and L1210 cells (Figure 1A). In addition, even at a dose of 20 μM, 20% L1210 and 40% HPB-ALL cells remained Annexin V^-^ and PI^-^ (Figure 1A). The marked differences in As_2_O_3_ sensitivity among the T-cell lines can also be shown by calculating the concentration of As_2_O_3_ that killed 50% of the cells (IC_50_ value). The IC_50_ value of As_2_O_3_ was 1.5 μM for EL-4, 2.5 μM for BW5147, 6 μM for Jurkat, 13 μM for L1210 and 15 μM for HPB-ALL cells compared with 1.5 μM for the highly sensitive APL-derived NB4 cells (Figure 1A). As_2_O_3_ cytotoxicity was also evaluated through flow cytometric analysis of physical characteristics of As_2_O_3_-treated T-cell lines using forward (FSC) and side (SSC) scatters since dying or dead cells have lower FSC and higher SSC than living cells. Depending upon the dose of As_2_O_3_, two populations were observed on the dot plots for FSC *vs*. SSC. The one containing large cells (region R2) encompassed live cells, and the other containing smaller cells (region R1) encompassed dying/dead cells (Additional file 1: Figure S1A). We observed that 50% of the cells of region R2 shifted to region R1 at 2.5 to 3 μM As_2_O_3_ for BW5147 and EL-4 cells, 6 μM for Jurkat cells, and 15 μM for L1210 and HPB-ALL cells compared with 1.5 μM for the APL cell line NB4 (Figure 1B). In parallel, we determined As_2_O_3_-induced cell growth inhibition by counting the total cell number in each condition using flow cytometry. IC_50_ value for growth inhibition was around 1 μM for NB4 and EL-4 cells, 3.5 μM for BW5147 cells, 8 μM for Jurkat, 15 μM for L1210 and HPB-ALL cells. In summary, EL-4 and BW5147 cells, and to a lesser extent Jurkat cells, are among the most sensitive T-cell lines to As_2_O_3_-induced cell growth inhibition and cytotoxicity, whereas L1210 and HPB-ALL cells are the most resistant.

### Lack of evidence for the implication of GSH and O_2_^-^ in As_2_O_3_-induced cytotoxicity in T-cell lines

**Figure 2:**
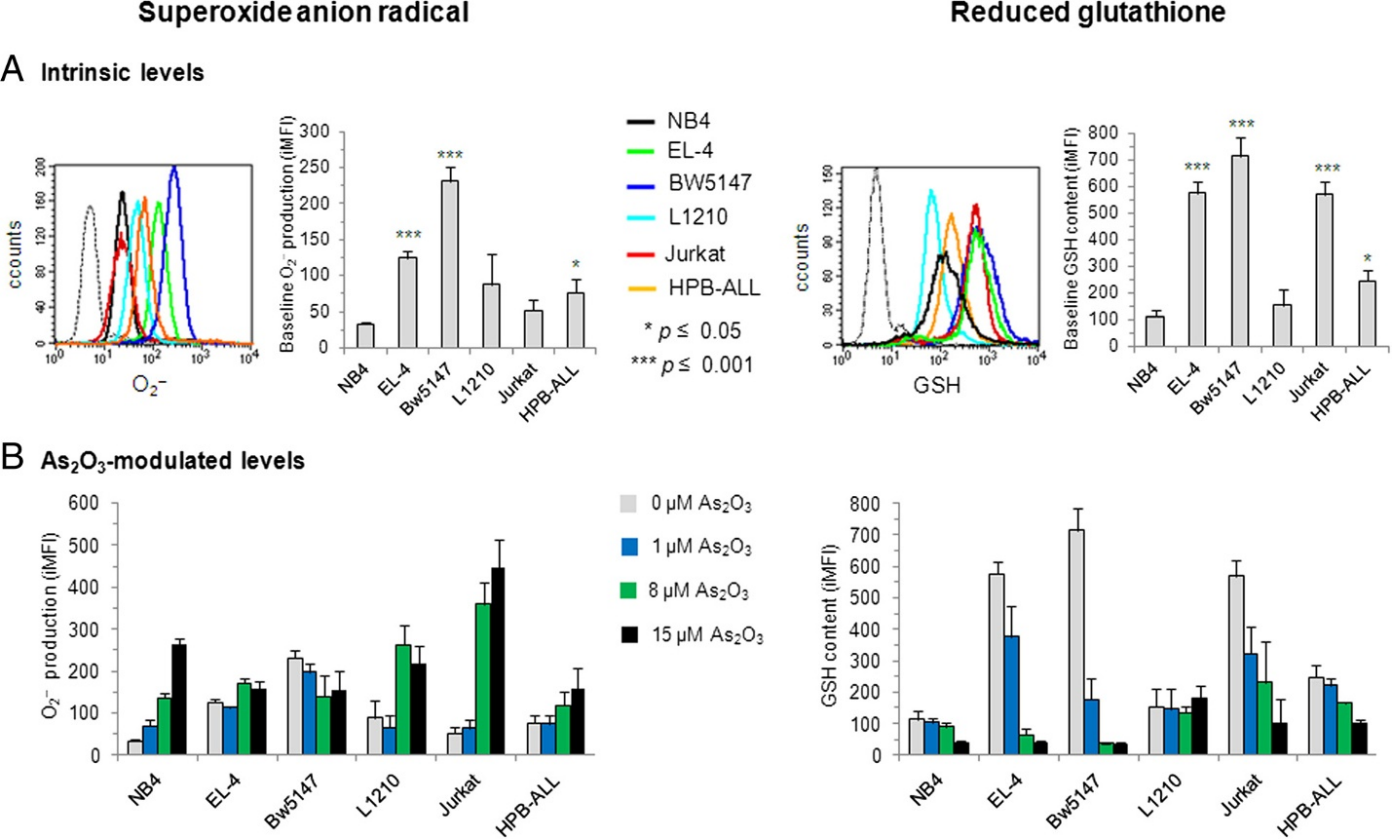
**Intracellular levels of O**_**2**_^**-**^ **and GSH in cell lines before and after As**_**2**_ **O**_**3**_ **treatment.** Intracellular levels of O_2_^-^ and GSH were measured using CMFDA or DHE fluorescent probes and flow cytometry. **(A)** Untreated APL-derived NB4 cells and T-cell lines were examined for intrinsic levels of O_2_^-^ production and GSH content. Histograms obtained with specific fluorescent probes (colored histograms) are overlaid on fluorescence histograms of unstained cells (black dotted histogram). A total of 20,000 events were analyzed for each histogram. Graphs report mean ± SE (n = 3 independent experiments) of integrated MFI values for O_2_^-^ production and GSH content. The integrated MFI is calculated by multiplying the frequency of positive cells by the MFI of O_2_^-^ and GSH. Asterisks denote statistically significant differences for levels of GSH or O_2_^-^ between leukemic T-cell lines and APL-derived NB4 cells: *p ≤0.05; ***p ≤0.001. **(B)** O_2_^-^ production and GSH content were examined in APL-derived NB4 cells and T-cell lines treated with 1 μM to 15 μM As_2_O_3_ for 24 h. Graphs report mean ± SE (n = 3 independent experiments) of integrated MFI values for O_2_^-^ production and GSH content.

As_2_O_3_ is able to impair the function of the mitochondrial respiratory chain, leading to increased O_2_^-^ production [24]. O_2_^-^ is neutralized by intracellular GSH, which is the major antioxidant produced by the cell. The detoxification function of intracellular GSH also includes the cellular efflux of As_2_O_3_ [25]. Therefore, we performed flow cytometry analysis of intracellular GSH content and O_2_^-^ production in the T-cell lines before and after treatment with As_2_O_3_ (Figure 2). APL-derived NB4 cells were used as a reference because intracellular redox status has been shown to be important in APL sensitivity to As_2_O_3_ [9, 10]. As observed in many cancer cells [25], the untreated cell lines showed considerable heterogeneity in the intrinsic levels of O_2_^-^ production, with untreated NB4 cells displaying the lower level of O_2_^-^ (Figure 2A). In As_2_O_3_-treated APL-derived NB4 cells, O_2_^-^ production increased with the dose of arsenic (Figure 2B). In As_2_O_3_-sensitive EL-4 and BW5147 T-cell lines, O_2_^-^ production was not increased by As_2_O_3_ treatment even at the high dose of 15 μM (Figure 2B). In contrast, in As_2_O_3_-resistant Jurkat and L1210 T-cell lines, O_2_^-^ production was markedly increased (Figure 2B). Therefore, these data indicate that the differences in As_2_O_3_ sensitivity between EL-4, BW5147, Jurkat and L1210 cells are not directly linked to increased production of O_2_^-^.

As expected, As_2_O_3_-sensitive APL-derived NB4 cells displayed extremely low level of intrinsic GSH content (Figure 2A). In contrast, the intrinsic GSH content in untreated EL-4 and BW5147 T-cell lines were significantly higher than in untreated HPB-ALL and L1210 T-cell lines (Figure 2A), although L1210 and HPB-ALL cells were considerably more resistant to the cytotoxic effect of As_2_O_3_ than EL-4 and BW5147 cells. At the dose of 1 μM As_2_O_3_, levels of GSH were unaffected by the treatment in the extremely sensitive NB4 cells, but were markedly decreased in As_2_O_3_-sensitive BW5147 T cells and also, although to a lesser extent, in the As_2_O_3_-resistant Jurkat cells (Figure 2B). Furthermore, in the extremely resistant L1210 T cells, GSH production was unchanged even at the high dose of 15 μM As_2_O_3_ (Figure 2B). Therefore, our data indicate that intracellular GSH content alone cannot account for the differences in As_2_O_3_ sensitivity observed among the five T-cell lines.

### Induction of B220/CD45R expression on As_2_O_3_-treated human and murine T-cell lines

**Figure 3:**
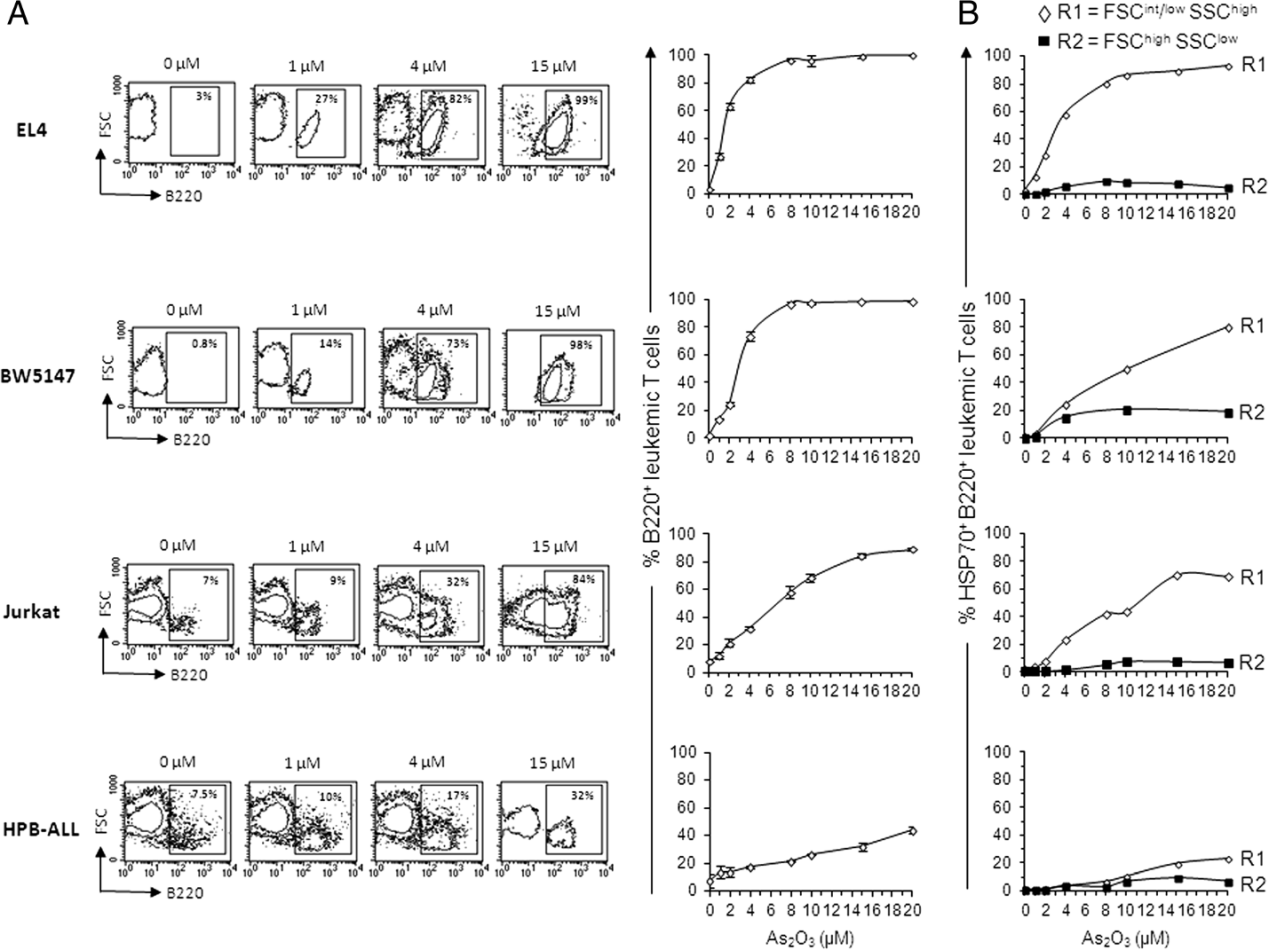
**Induction of phosphatase B220/CD45R and HSP70 on As**_**2**_ **O**_**3**_ **-treated T-cell lines.** EL-4, BW5147, Jurkat, CD45-deficient Jurkat variant (J45.01) and HPB-ALL T-cell lines were treated with As_2_O_3_ for 24 h in doses ranging from 1 to 20 μM. **(A)** T-cell lines were then stained with PE-conjugated anti-B220/CD45R mAb or PE-conjugated rat IgG2a isotype control, and then analyzed by flow cytometry with respect to size (FSC) and B220 expression. FSC vs. B220 contour plots were used to determine the percentage of B220^+^ cells in region R1 (FSC^int/low^SSC^high^). At least 20,000 events were analyzed per sample. Squares in contour dots contain B220^+^ cells, and are drawn relative to the location of the cells in FSC vs. IgG2a isotype control contour plots. Numbers in the squares indicate the percentages of B220^+^ cells. Graphs report mean percentages ± SE (n =10 independent experiments) of B220^+^ cells versus the dose of As_2_O_3_. **(B)** T-cell lines were stained with APC-conjugated anti-B220 and FITC-conjugated anti-HSP70 antibodies or FITC-conjugated IgG and APC-conjugated IgG2a isotype control. FSC vs. SSC dot plots were used to define gates R1 and R2 with FSC^int/low^SSC^high^ and FSC^high^SSC^low^, respectively. HSP70 vs. B220 dot plots were then gated in R1 and R2 to determine the percentages of cells expressing HSP70, B220 or both. At least 20,000 events were analyzed for each sample. Graphs report the percentages of cells co-expressing B220 and HSP70 in gates R1 (◊) or R2 (■) versus the dose of As_2_O_3_.

Normal effector T cells undergoing apoptosis at the end of an immune response express B220 on their cell surface. These apoptotic B220^+^ T cells are solely found within the FSC^int/low^ SSC^high^ subset by flow cytometry analysis [15, 16]. Interestingly, we observed that As_2_O_3_ is also able to induce cell-surface expression of B220 on EL-4, BW5147, Jurkat and HPB-ALL T-cell lines in a dose-dependent manner (Figure 3). However, B220 expression levels differed significantly among these four T-cell lines. Thus, a significantly higher percentage of EL-4 cells, and to a lesser extent of BW5147 and Jurkat cells, expressed B220 compared to HPB-ALL cells (for instance, see 2 μM dose in Table 1 or 4 μM dose in Figure 3). B220^+^ leukemic T cells were found in both gate R1 and R2 that contained FSC^int/low^ SSC^high^ dying cells (Figure 3) and FSC^high^ SSC^low^ living cells (data not shown), respectively, although the numbers were significantly higher in gate R1. As_2_O_3_ also induces B220 expression at the mRNA level in a dose- and time-dependent manner. High levels of B220 mRNA were found in EL-4 and BW5147 within 3 h of treatment with 1 μM As_2_O_3_. Higher doses of As_2_O_3_ (e.g., 4 μM for 3 h) or longer treatment (e.g., 1 μM for 9 h) resulted in increased levels of B220 mRNA (Additional file 1: Figure S1C). In contrast, As_2_O_3_ did not induce B220 expression on APL-derived NB4 cells (data not shown). To confirm that A_2_O_3_-induced cytotoxicity in leukemic T cells and B220 expression are mechanistically related, we analyzed CD45-deficient Jurkat cells (clone J45.01) for their As_2_O_3_ sensitivity over a large range of doses (1 to 20 μM). As shown in Additional file 1: Figure S1B, J45.01 T-cell line displays a strong reduction in the expression level of CD45 per cell (MFI) compared to wild-type Jurkat cells (MFI J45.01: 40; MFI Jurkat: 1528; MFI isotype control: 5). The IC_50_ value for cytotoxicity was around 12 μM for J45.01 compared to 6 μM for wild-type Jurkat cells, corroborating the critical role of the B220 isoform of CD45 in the resistance/sensitivity of leukemic T cells to As_2_O_3_.

**Table 1:** **Opposite effects of As**_**2**_ **O**_**3**_ **or Calcium ionophore A23187 on a panel of leukemic T-cell lines**

	As _2_O _3_ (2 μM*)				Calcium ionophore A23187 (100 nM)			
T-cell lines	B220 upregulation**	CD69 upregulation**	HSP70 upregulation**	Cell death***	B220 upregulation**	CD69 upregulation**	HSP70 upregulation**	Cell death***
**EL-4**	63	No	35	70	7	Yes	no	1
**BW5147**	30	No	20	39	19	Yes	no	25
**Jurkat clone E6-1**	22	No	10	12	30	Yes	no	28
**HPB-ALL**	10	No	3	5	80	Yes	no	75
**L1210**	Constitutive expression	No	5	10	Constitutive expression	Yes	no	55

*a clinically relevant concentration of As_2_O_3._

**percentage of positive cells determined by flow cytometry.

***percentage of PI^+^ cells (either Annexin V^-^ or Annexin V^+^) determined by flow cytometry.

Unexpectedly, we also found that L1210 T cells constitutively express B220 at the plasma membrane. L1210 cells undoubtedly belong to the T-cell lineage because they coexpress the mouse pan T-cell marker CD90 (Additional file 2: Figure S2A). To the best of our knowledge, we have shown for the first time that B220 can be constitutively expressed at high levels on T cells, in the absence of external stimulation. As_2_O_3_ treatment did not change either the percentages of B220^+^ L1210 cells, or the expression level of B220 per cell (MFI) (Additional file 2: Figure S2B).

**Figure 4:**
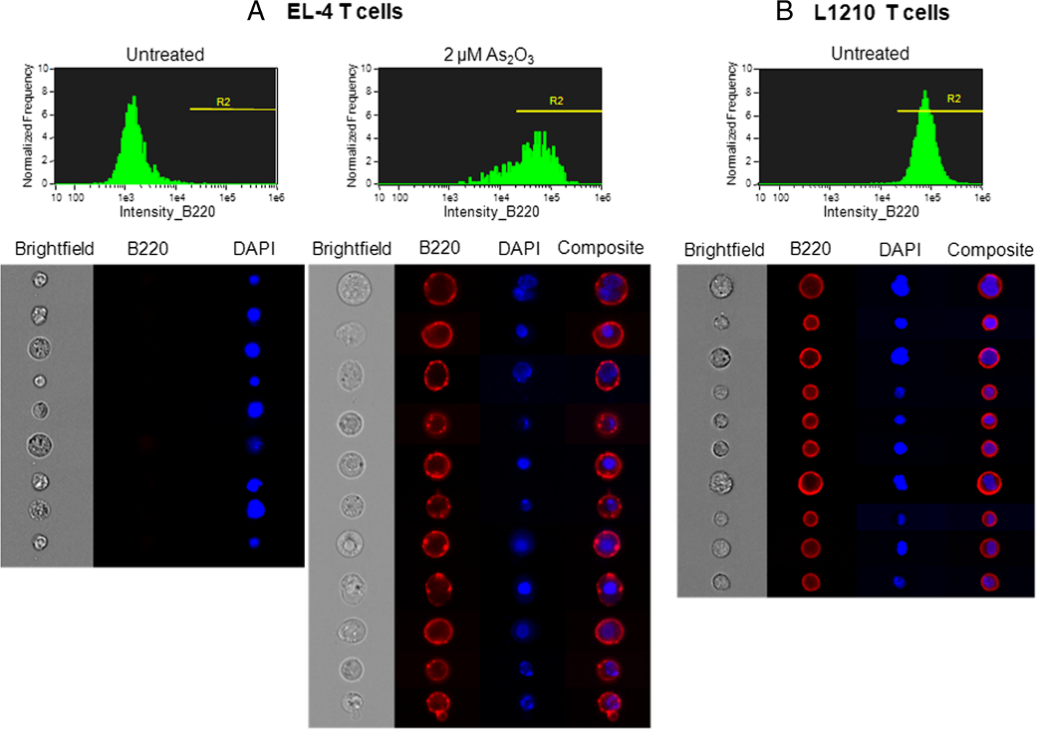
**Subcellular localization of constitutively expressed and As**_**2**_ **O**_**3**_ **-induced B220/CD45R molecules.** Untreated and As_2_O_3_-treated (2 μM for 24 h) EL-4 cells **(panel A)**, and untreated L1210 cells **(panel B)** were stained with PE-conjugated anti-B220/CD45R mAb and DAPI nuclear dye. Samples were then analyzed on the ImageStream system. At least 10,000 images were collected per sample at 40× and 60× magnification. B220-PE positive cells were gated (gate R2) according to the principle for flow cytometry as shown on histogram. Background staining was determined using PE-conjugated rat IgG2a isotype control. Representative cell images of brightfield illumination, B220-PE (red) and DAPI (blue) fluorescence and composite images of B220/DAPI are shown on panel **A** and **B**.

Furthermore, we have compared the subcellular localization and distribution of B220 molecules that were either constitutively expressed on L1210 cells or As_2_O_3_-induced on EL-4 cells, using ImageStream device, which allows simultaneous flow cytometry and fluorescence microscopy analysis on a large numbers of cells. Whole cell and nuclear boundaries were identified using brightfield image and DAPI staining, respectively. In the absence of As_2_O_3_ treatment, no labeling with anti-B220/CD45R mAb was observed in EL-4 cells (Figure 4A). In contrast, in untreated L1210 cells and 2 μM As_2_O_3_-treated EL-4 cells, B220 molecules were expressed at high levels with a restricted localization to the cell surface (Figure 4A and B), confirming the results obtained by flow cytometry. However, B220 molecules appeared less uniformly distributed over the plasma membrane of EL-4 cells (Figure 4A) than L1210 cells (Figure 4B). Thus, L1210 cells showed a uniform fluorescence at the periphery of the cell, whereas a variable number of bright spots of fluorescence were distributed at the periphery of EL-4 cells.

### Duration of B220/CD45R membrane expression after As_2_O_3_ treatment

To determine the duration of B220 expression following As_2_O_3_ treatment, EL-4 and Jurkat cells were cultured in the presence of 1 to 8 μM As_2_O_3_ for 24 h. Cells were then extensively washed to eliminate As_2_O_3_, and cultured in complete medium for 9 additional days. As_2_O_3_ treatment for 24 h induced B220 expression in a dose-dependent manner (Additional file 3: Figure S3, graphs), particularly on EL-4 cells, corroborating the findings reported in Figure 3. On day 1 post-treatment, and at concentrations of 1 μM and 8 μM As_2_O_3_, the percentages of B220^+^ EL-4 cells were around 50% and 95%, respectively. One week later, the percentage of B220^+^ EL-4 cells fell below 15% at 1 μM As_2_O_3_, whereas it remained at 90% at the concentration of 8 μM (Additional file 3: Figure S3, graphs). Likewise in Jurkat cells, on day 7 post-treatment, around 50% of cells treated with 8 μM As_2_O_3_ expressed B220 (Additional file 3: Figure S3, dot plots and graph), while B220 could no longer be detected on Jurkat cells treated with 1 to 4 μM As_2_O_3_ (Additional file 3: Figure S3, graph). The loss of B220 expression on EL-4 and Jurkat cells was accompanied by a decreased cell number in gate R1 (FSC^int/low^ SSC^high^) and an expansion of viable cells in gate R2 (Additional file 3: Figure S3, dot plots, see days 7 and 9). The duration of B220 expression in gate R1 was dependent on the dose of As_2_O_3_ treatment. Thus, a longer duration of B220 expression was associated with a higher concentration of As_2_O_3_, and a higher number of B220^+^ cells at the time of As_2_O_3_ removal. The decrease in the percentages of B220^+^ cell population could be due to the death of B220^+^ cells, and/or the proliferation of the remaining B220^-^ cells. However, even on day 9 post-treatment, EL-4 and Jurkat cells had not fully recovered their initial proliferative capacity (data not shown). Our data suggest that dead B220^+^ T cells release their As_2_O_3_ content into the medium, which is subsequently recaptured by living B220-negative T cells. The amount of released As_2_O_3_ is dependent on the dose used at the time of treatment.

### Expression of membrane-bound HSP70 on As_2_O_3_-treated T-cell lines parallels the induction of B220/CD45R

Heat shock protein 70 (HSP70) is upregulated intracellularly in respond to a variety of stresses including chemical or chemotherapeutic agents. HSP70 proteins can also be found at the cell surface of some tumor cells. Notably, membrane-bound HSP70 has been shown to elicit antitumor immunity [26, 27]. Therefore, HSP70 and B220 expression was determined by flow cytometry on the plasma membrane of the T-cell lines treated with As_2_O_3_ for 24 h in doses ranging from 1 to 20 μM. HSP70 was not detected on untreated T-cell lines (Figure 3B). As_2_O_3_ treatment upregulated membrane-bound HSP70 in EL-4, BW5147, Jurkat and HPB-ALL cells in a dose-dependent manner. The expression pattern of HSP70 was parallel to that of B220, and HSP70 was expressed in B220^+^ cells only (Figure 3B). As observed for B220 expression, a significantly higher percentage of EL-4 cells, and to a lesser extent of Jurkat cells, than HPB-ALL cells expressed HSP70 (Figure 3B) (Table 1). L1210 cells, constitutively expressing B220, had a low level of expression of membrane-bound HSP70 (Additional file 2: Figure S2C) similar to HSP70 levels observed in HPB-ALL cells. Therefore, As_2_O_3_ treatment induced both HSP70 and B220 membrane expression and the levels of expression correlated with leukemic T-cell lines sensitivity to As_2_O_3_.

### B220/CD45R expression on As_2_O_3_-treated leukemic T cells is not a direct consequence of T-cell activation unlike the expression induced by the calcium ionophore A23187

The induction of B220 on EL-4, BW5147, Jurkat and HPB-ALL leukemic T cells treated with As_2_O_3_ is suggestive of the induction observed on normal T cells entering apoptosis after repeated activation by their antigen. T-cell activation can be evidenced by measuring the expression of surface marker CD69 [28]. Therefore, we have analyzed the induction of CD69 on the T-cell lines treated with As_2_O_3_ as well as with PMA and calcium ionophore A23187 as positive controls. As expected, the activation of leukemic T cells with 10 ng/ml of PMA for 5 h induced a strong expression of the activation marker CD69 in cells within region R2 only, which encompasses living cells. In contrast, we did not detect the expression of CD69 by flow cytometry on the cell surface of As_2_O_3_-treated leukemic T cells, whatever the doses tested (1 to 20 μM, data not shown), suggesting that the induction of B220 expression by As_2_O_3_ is not a direct consequence of T-cell activation.

**Figure 5:**
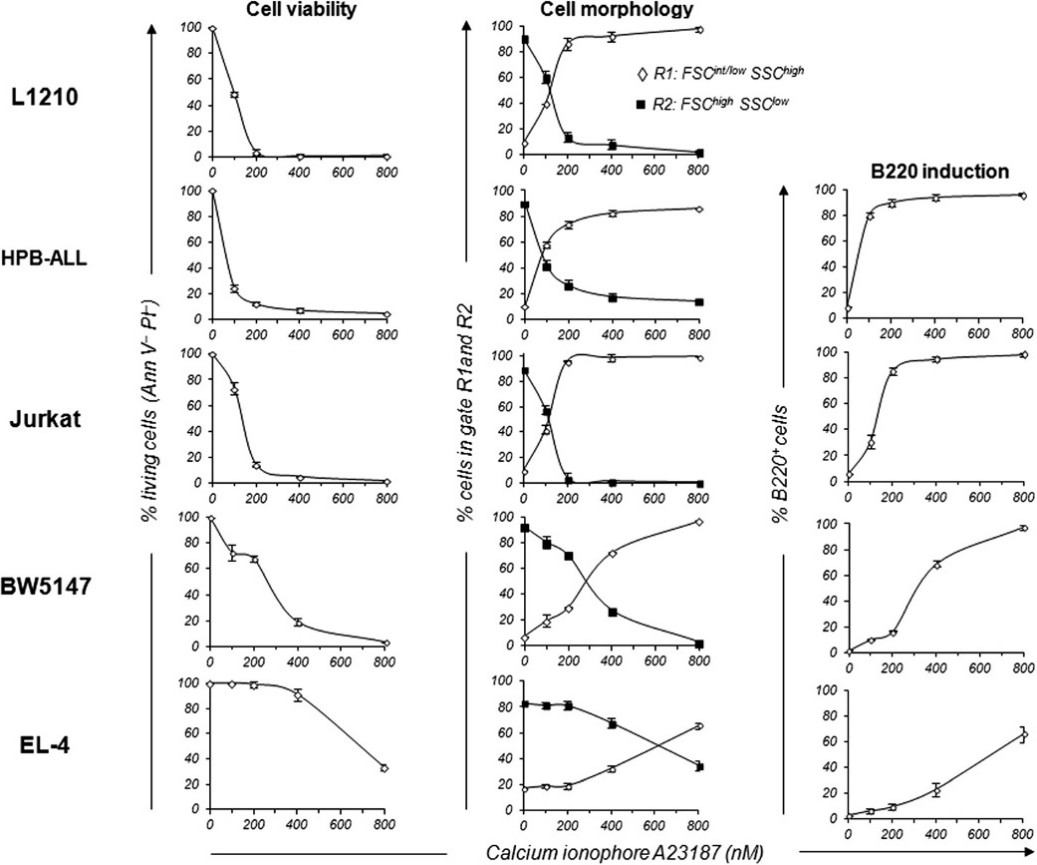
**Cell viability, cell morphology and B220/CD45R induction in T-cell lines stimulated with calcium ionophore A23187.** Jurkat, HPB-ALL, L1210, EL-4 and BW5147 cells were treated with Calcium ionophore A23187 for 48 h in doses ranging from 100 to 800 nM. *Cell viability measurement*. Untreated and A23187-treated cells were stained with Annexin V and PI, and the percentages of living cells (Ann V^-^ PI^-^) determined by flow cytometry. Graphs show mean percentages ± SE of Ann V^-^ PI^-^ from 4 independent experiments. *Cell morphology analysis*. Untreated and A23187-treated cells were analyzed by flow cytometry with respect to size (FSC) and granulosity (SSC). Regions R1 (◊) and R2 (■) identified on FSC vs. SSC dot plots encompassed cells with FSC^int/low^SSC^high^ and FSC^high^SSC^low^, respectively. *B220 induction*. Untreated and A23187-treated T-cell lines were stained with anti-B220/CD45R mAb and analyzed by flow cytometry. Graphs report mean percentages ± SE (n = 4 independent experiments) of B220^+^ EL-4, BW5147, Jurkat and HPB-ALL cells at the indicated concentration of A23187. At least 20,000 events were analyzed per sample in all experiments reported in the graphs.

As observed with PMA, activation of the leukemic T-cell lines with 100 nM to 800 nM of the calcium ionophore A23187 for 24 h induced the expression of CD69 on EL-4, BW5147, L1210, and Jurkat cells and also, to a lesser extent, on HPB-ALL cells (Table 1). However, the number of CD69^+^ cells decreased with the dose of A23187, and hardly any CD69^+^ cells were detected at the higher dose of A23187 (data not shown). In contrast, a dose-dependent expression of B220 was detected on EL-4, BW5147, Jurkat and HPB-ALL cells, although significant differences were observed in the percentages of B220^+^ cells among T-cell lines (Figure 5). Thus, 50% of B220^+^ cells were detected at a dose of A23187 around 50 nM for HPB-ALL cells, 100–130 nM for Jurkat cells, 275 nM for BW5147 and 600 nM for EL-4 cells (Figure 5, see B220 induction). Interestingly, at around the same dose of A23187 that induced B220 expression, 50% of HPB-ALL, Jurkat, BW5147 and EL-4 cells died (Annexin V and PI staining, Figure 5, see Cell viability) or shifted from region R2 to R1 on the FSC *vs*. SSC dot plots (Figure 5, see Cell morphology). In contrast to As_2_O_3_, the constitutive expression of B220 on L1210 T cells renders these cells remarkably sensitive to A23187 (Figure 5, see Cell viability and Cell morphology). Therefore, B220 expression level strictly correlates with the susceptibility of T cells to die following A23187 activation, suggesting an interesting link between transmembrane phosphatase B220 expression and cell death. Moreover, in contrast with As_2_O_3_ treatment, membrane-bound HSP70 was not upregulated following treatment with calcium ionophore A23187, even on the B220^+^ T-cell subpopulations (data not shown).

### Relative contributions of apoptosis and necrosis in T leukemia cell death induction by As_2_O_3_ or calcium ionophore A23187

**Figure 6:**
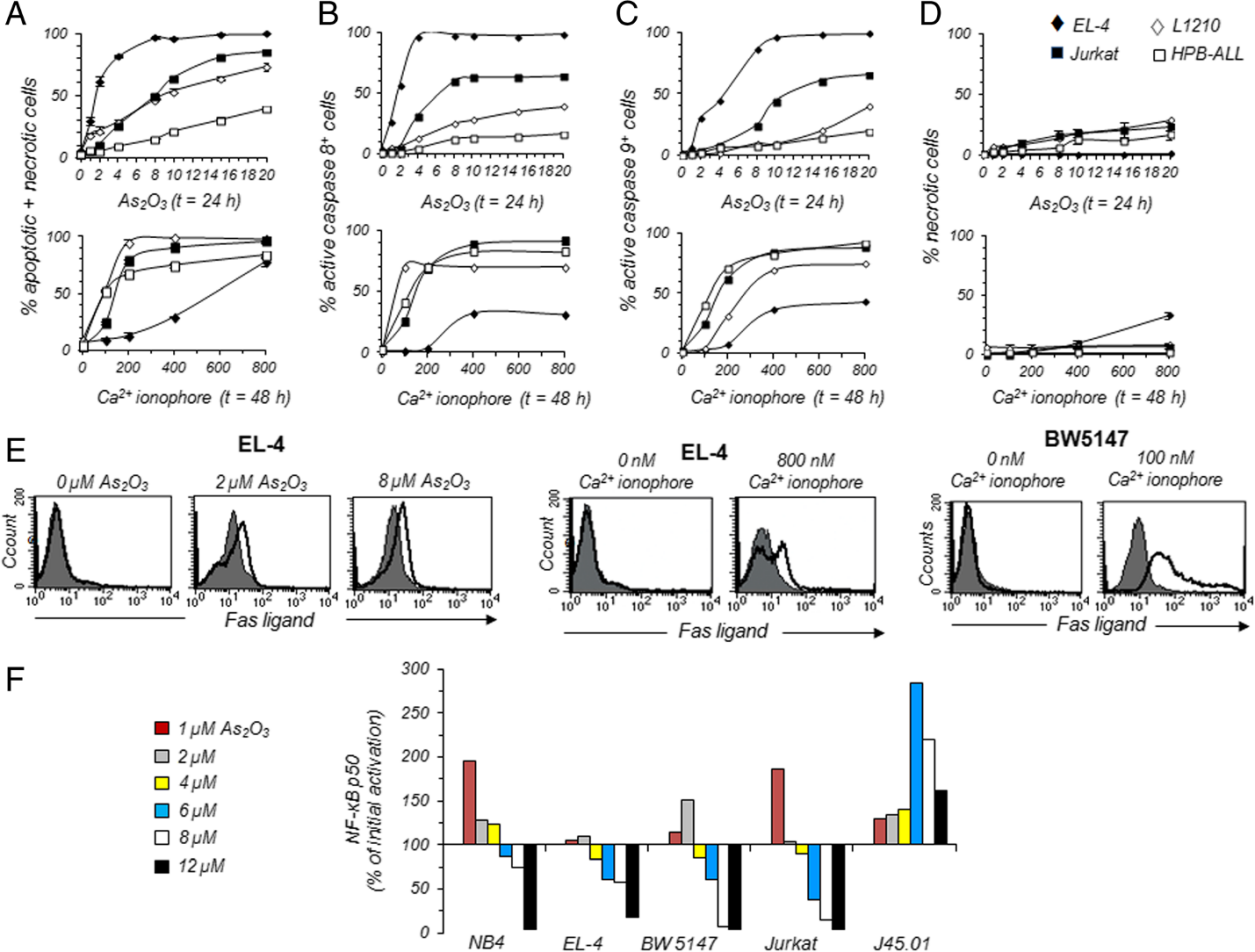
**Contribution of apoptosis, necrosis and NF-κB p50 pathways in the cytotoxicity of As**_**2**_ **O**_**3**_ **or calcium ionophore A23187. (A to E)** EL-4 (♦), L1210 (◊), Jurkat (■) and HPB-ALL (□) T-cell lines were treated with 1 to 20 μM As_2_O_3_ for 24 h or 100 to 800 nM A23187 for 48 h. Cells treated with As_2_O_3_ or A23187, or left untreated, were subsequently stained with PI and CaspaTag Caspase 8 or Caspase 9 fluorescein in situ fluorescence assay kit. The stained cells were analyzed by flow cytometry in order to determine the percentages of dead cells (either apoptotic or necrotic cells, graphs **A**) (n = 5 independent experiments), the percentages of PI^+^ apoptotic cells containing active caspase 8 (graphs **B**) or active caspase 9 (graphs **C**), the percentage of necrotic cells (graphs **D**). Human or murine FasL expression was detected using anti-FasL mAb and flow cytometry (histograms **E**). Histograms obtained with PE-conjugated Armenian hamster (clone MFL3) or mouse (clone NOK-1) anti-FasL mAb (open histogram) are overlaid on histograms obtained with PE-conjugated Armenian hamster or mouse IgG1 isotype control (shaded histogram) (n = 3 independent experiments). At least 20,000 events were analyzed for each sample. **(F)**. Nuclear extracts were prepared from NB4, EL-4, BW5147, Jurkat and J45.01 cells treated with 1 to 12 μM As_2_O_3_ for 24 h. Levels of NF-κB p50 in 5 μg of nuclear extracts were quantified by a DNA binding ELISA assay. Results are representative of two other experiments.

It is widely considered that As_2_O_3_ exerts its cytotoxic activity by triggering cell apoptosis [12]. However, it has been reported that As_2_O_3_ can induce cell death independently of caspase activity [29, 30]. We have determined whether the apoptotic and/or necrotic pathways were activated upon As_2_O_3_ or calcium ionophore A23187 treatment in EL-4, Jurkat, HPB-ALL and L1210 cells. Because BW5147 cells displayed similar sensitivity to As_2_O_3_ and A23187 as EL-4 and Jurkat cells, respectively, the latter two T-cell lines were preferentially chosen for subsequent experiments. In the presence of As_2_O_3_ or A23187, no PI^+^ cells (either Annexin V^-^ or Annexin V^+^) were detected in the B220-negative cell subpopulation (data not shown). In contrast, in the B220^+^ cell subpopulation, the IC_50_ value of As_2_O_3_ that causes either apoptotic or necrotic cell death was 1.5 μM for EL-4 cells, 8 μM for Jurkat cells and 9 μM for L1210 cells. For B220^+^ HPB-ALL cells, even at the higher dose of 20 μM As_2_O_3_, only 39 ± 2.5% of the cells were killed (Figure 6A). Furthermore, to discriminate between late apoptosis (PI^+^/Annexin V^+^) and necrosis (PI^+^/Annexin V^-^), we analyzed the levels of active initiator caspases 8 and 9 in the B220^+^ PI^+^-cell subpopulation. Significative differences were observed between the T-cell lines, even though the levels of active caspase 8 and/or 9 increased with the dose of As_2_O_3_ (Figure 6B and C). Thus, EC_50_ for caspase 8 activation was 2 μM for EL-4 cells and 6.5 μM for Jurkat cells (Figure 6B). To activate caspase 9 in these T-cell lines, almost twice as much As_2_O_3_ was required compared with caspase 8 (Figure 6C). Even at the higher dose of 20 μM As_2_O_3_, only 16 to 19% of HPB-ALL cells and ~40% of L1210 cells contained active caspase 8 or caspase 9 (Figure 6B and C). Since As_2_O_3_ can also induce cell death independently of caspase activity [30], we have determined the percentages of necrotic cells (PI^+^/Annexin V^-^ or PI^+^/active Caspase^-^). No necrotic cells were detected in As_2_O_3_-treated EL-4 cells. In contrast, 4 to 29% necrotic cells were detected in As_2_O_3_-treated Jurkat, HPB-ALL and L1210 cells (Figure 6D). In agreement with Figure 4, the IC_50_ value of calcium ionophore A237187 that cause either apoptotic or necrotic cell death was between 80 nM and 150 nM for L1210, HPB-ALL and Jurkat cells compared with around 600 nM for EL-4 cells (Figure 6A). The relative contributions of apoptotic and necrotic cell death in calcium ionophore A23187 cytotoxicity showed an opposite pattern compared with As_2_O_3_ cytotoxicity. In the presence of calcium ionophore A23187, up to 32% necrotic cells were detected in EL-4 cells, whereas no necrotic cells were detected in HPB-ALL cells and less than 8% were detected in L1210 and Jurkat cell lines (Figure 6D). Moreover, EL-4 cells did not contain active caspase 8 and 9, whereas high levels of active caspase 8 and 9 were detected in L1210, HPB-ALL, and Jurkat T-cell lines (Figure 6B and C).

To summarize, when high levels of B220 membrane expression were achieved with low doses of As_2_O_3_ or calcium ionophore A23187, the leukemic T-cell lines died by apoptosis only. When high doses of As_2_O_3_ or calcium ionophore A23187 were required to induce B220 expression, the T-cell lines died by both apoptosis and necrosis. The constitutive expression of B220 on L1210 cells did not favor apoptosis or necrosis, and cells died by a combination of the two. Taken together, our data suggest that transmembrane tyrosine phosphatase B220 plays a checkpoint role in apoptotic pathways since its expression is markedly and rapidly induced on the surface of T cells undergoing apoptosis.

### Expression of Fas and Fas ligand upon treatment with As_2_O_3_ or A23187

The presence of active initiator caspase 8 in the T-cell lines treated with A23187 or As_2_O_3_ suggested that cells entered apoptosis upon engagement of death receptors such as Fas. In normal T cells, Fas receptor is constitutively expressed whereas its ligand, FasL, is expressed after T cell activation. The five T-cell lines were treated or not with As_2_O_3_ or calcium ionophore A23187 for 24 h and 48 h, and the levels of Fas and FasL expression were determined by flow cytometry. Fas receptor is strongly expressed on untreated Jurkat cells, weakly expressed on untreated HPB-ALL cells, and not detected on untreated EL-4, BW5147 and L1210 cells. No significant modulation in Fas receptor expression was observed after treatment with As_2_O_3_ or A23187 (data not shown). Its ligand, FasL, is not expressed on untreated T-cell lines. Upon treatment with 2 μM As_2_O_3_ for 24 h, only the highly As_2_O_3_-sensitive EL-4 T-cell line expressed FasL, with 100% of EL-4 cells being FasL positive although weakly (Figure 6E). FasL^+^ EL-4 cells were equally detected in regions R1 and R2 (data not shown), suggesting that FasL is expressed before apoptosis. FasL-expressing BW5147, L1210, Jurkat and HPB-ALL cells were not detected even after 48 h of treatment with As_2_O_3_ (data not shown).

Treatment with the calcium ionophore A23187 upregulated FasL expression on EL-4, BW5147 and L1210 cells (Figure 6E and Additional file 2: Figure S2D), but not on Jurkat and HPB-ALL cells (data not shown). While 24 h of treatment were sufficient to induce FasL expression on BW5147 and EL-4 cells, 48 h were required to induce such expression on L1210 cells (Additional file 2: Figure S2D). However, the expression levels of FasL were significantly higher on BW5147 cells than on EL-4 and L1210 cells. In addition, FasL upregulation was achieved using a concentration of calcium ionophore A23187 around 4 and 8 times higher in EL-4 cells and L1210 cells, respectively, than in BW5147 cells. FasL-expressing EL-4, BW5147 and L1210 cells were equally detected in regions R1 and R2 (data not shown). The expression of FasL on EL-4 cells treated with As_2_O_3_ or A23187, and on BW5147 and L1210 cells only when treated with A23187, strongly suggest that As_2_O_3_ and A23187 trigger FasL expression through different signaling pathways. Furthermore, the absence of either Fas or FasL, or both, on A_2_O_3_- or A23187-treated cells indicates that their activation of caspase 8 is independent of the Fas/FasL pathway and must depend on other death pathway.

### As_2_O_3_ treatment represses nuclear translocation of NF-κB p50

NF-κB activity regulates the apoptosis of various cancer cell lines. Activation of NF-κB can promote or prevent apoptosis, depending on the stimuli utilized and the cell type [31]. Conflicting results have been published on the activation status of NF-κB in As_2_O_3_-treated leukemia T cells. One report has shown that As_2_O_3_ treatment markedly decreases constitutive NF-κB activation [32]. In another study, the authors could not detect any decrease in the translocation of the p65 subunit of NF-κB [33]. This discrepancy may be due to differences in the doses of As_2_O_3_ used to treat leukemic T cells. Therefore, we have quantified NF-κB p50 in nuclear extracts from NB4, EL-4, BW5147, Jurkat (clone E6-1) and CD45-deficient variant (clone J45.01) treated with a large range of doses (i.e. 1 μM to 12 μM). We found that As_2_O_3_ treatment represses nuclear translocation of NF-κB p50 in As_2_O_3_-treated T cells in a dose-dependent manner (Figure 6F). Importantly, nuclear translocation of NF-κB p50 was strongly increased in the CD45-deficient Jurkat T-cell line (clone J45.01) treated with As_2_O_3_ compared to wild-type Jurkat cells (Figure 6F). Thus, after a treatment with 6 μM As_2_O_3_ (IC_50_ value for the cytotoxic effect of As_2_O_3_ on Jurkat cells) J45.01 cells contained markedly more nuclear NF-κB p50 (about 3 times more) than wild-type Jurkat cells, suggesting a link between B220 and NF-κB signaling pathways.

## Discussion

In APL-derived NB4 cells, As_2_O_3_ triggers apoptosis at concentrations of 0.5 to 2 μM [8]. The cytotoxic properties of As_2_O_3_ are not restricted to APL, and As_2_O_3_ induces apoptosis in various types of hematopoietic and solid tumors [12]. However, hematologic tumor cells vary considerably in their sensitivity to As_2_O_3_. To gain insight into the mechanisms underlying the As_2_O_3_ sensitivity of malignant T cells, we firstly selected two human (Jurkat and HPB-ALL) and three murine (EL-4, BW5147 and L1210) T-cell lines for their marked differences in sensitivity to As_2_O_3_ cytotoxicity over a large range of doses (i.e. 1 μM to 20 μM). Thus, 50% of EL-4 cells are killed at a clinically relevant concentration of about 1–2 μM As_2_O_3_, whereas concentrations of ~2.5 μM As_2_O_3_ for BW5147, ~6 μM As_2_O_3_ for Jurkat and ~12–15 μM As_2_O_3_ for L1210 and HPB-ALL T-cell lines are required to kill approximately 50% of the cells. Using this tumor panel, we have shown that: 1) differences in the intracellular levels of GSH and O_2_^-^ are not sufficient to account for their differences in As_2_O_3_ sensitivity; 2) transmembrane tyrosine phosphatase B220 is induced on EL-4, BW5147, Jurkat and HPB-ALL T-cell lines, but not APL-derived NB4 cells, upon treatment with As_2_O_3_ in a dose and time dependent manner; 3) the degree of B220 induction on the T-cell lines is strongly correlated with the sensitivity to As_2_O_3_ cytotoxicity; 4) surprisingly, B220 is constitutively expressed on the L1210 T-cell line; 5) membrane-bound HSP70, known to induce antitumor immunity, is upregulated by As_2_O_3_ in parallel with B220 induction; 6) initiator caspases 8 and 9 are activated by As_2_O_3_ in the T-cell lines where this activation parallels B220 induction, but not in L1210 cells; 7) As_2_O_3_ represses nuclear translocation of NF-κB p50 in a dose dependent manner; 8) FasL upregulation by As_2_O_3_ is found on Fas-negative EL-4 cells only, indicating that caspase 8 activation is most probably independent of the Fas/FasL pathway [34]. However, the absence of FasL on some T-cell lines (either human or murine) might be due to the rapid shedding of FasL by protease activities induced by As_2_O_3_ or calcium ionophore A23187, as we have reported for TNF-α and CD62L upon treatment with Ionomycin or ATP [35].

Contradictory data have been published on the efficacy of As_2_O_3_ treatment against tumors belonging to the lymphoid lineages. Evidence for a pro-apoptotic effect of As_2_O_3_ against human malignant T- and B-cell lines [36], cutaneous T-cell lymphoma [37] or HTLV-I-associated adult T-cell leukemia [38] was provided by in vitro studies. However, it has been shown, in a multi-institution phase II study, that As_2_O_3_ exhibits limited efficacy against lymphoid malignancies, even though the patients received ascorbic acid in addition to As_2_O_3_ [39]. The authors of this clinical study expected that agents such as ascorbic acid, which depletes intracellular GSH, could potentiate arsenic-induced apoptosis, since it has been described previously [10] that the sensitivity of cells to As_2_O_3_ are inversely correlated with their intracellular GSH content. As_2_O_3_ interacts with sulfhydryl (SH) groups of biologically active molecules. The binding of As_2_O_3_ to the SH group of GSH could cause a drastic decrease in the capacity to scavenge ROS in cells with a low basal GSH content, resulting in overproduction of intracellular ROS that could trigger cell apoptosis. However, our present study shows that EL-4 and BW5147 T-cell lines, which were the most sensitive to As_2_O_3_-induced cytotoxicity among the cells lines studied (IC_50_ of approximately 1 to 2.5 μM) had the highest baseline GSH content (Figure 2). Likewise, L1210 and HPB-ALL T cells that were significantly more resistant to As_2_O_3_ cytotoxicity (IC_50_ of approximately 12 to 15 μM) had significantly lower baseline GSH content than EL-4 and BW5147 cells (Figure 2). In the presence of As_2_O_3_, intracellular GSH content was differently modulated in the T-cell lines, but without any correlation with their As_2_O_3_ sensitivity. Thus, GSH level in the highly sensitive NB4 cell line was unaffected by the dose of 1 μM As_2_O_3_, whereas it was markedly decreased in the BW5147 T-cell line and also, although to a lesser extent, in the resistant Jurkat cells (Figure 2). Because intracellular GSH content did not appear to be a key factor in determining As_2_O_3_ sensitivity of these five leukemia T-cell lines, we further explored the evolution of O_2_^-^ production upon treatment with As_2_O_3_. The five T-cell lines showed a great heterogeneity in levels of intrinsic O_2_^-^ production (Figure 2), which, interestingly, were 2 to 8 times higher than in APL-derived NB4 cells. As expected, the levels of O_2_^-^ were significantly increased in APL-derived NB4 cells in the presence of As_2_O_3_. In T-cell lines, the situation was more complex. While the levels of O_2_^-^ increased significantly, in Jurkat, L1210, and to a lesser extent HPB-ALL T cells treated with As_2_O_3_, they remained unchanged in EL-4 and BW5147 T cells. Therefore, the changes of O_2_^-^ production upon treatment with As_2_O_3_ are not correlated with the differences in As_2_O_3_ sensitivity that we observed among the five T-cell lines. In contrast to other tumor cells [5], neither the intracellular GSH content nor the production of O_2_^-^ had any decisive effect on As_2_O_3_-induced apoptosis of the five T-cell lines studied. Therefore, our findings suggest that the depletion of leukemic T cells in their intracellular GSH content by ascorbic or butyric acid are not necessarily relevant to potentiate the cytotoxic effect of As_2_O_3_. Furthermore, it has been reported that the expression level of aquaglyceroporin (AQP)9, a transmembrane protein that controls arsenic transport, correlated positively with As_2_O_3_-induced cytotoxicity in myeloid and lymphoid leukemia cell lines [40]. In contrast, the overexpression of AQP9 in melanoma cells significantly increased the resistance to arsenite-induced apoptosis [41]. These reports prompted us to determine the levels of AQP9 mRNA by semi-quantitative RT-PCR in EL-4, BW5147, L1210, Jurkat and HPB-ALL T-cell lines, and in APL-derived NB4 cells. However, we did not find any correlation between the expression levels of AQP9 mRNA and the As_2_O_3_ sensitivity in the T-cell lines (unpublished data). Therefore, our data strongly suggest the existence of additional factors determining the sensitivity of T cells to As_2_O_3_ cytotoxicity. Herein, we have hypothesized that phosphatase B220 could be such a factor, since we have shown previously that treatment with As_2_O_3_ of autoimmune MRL/*lpr* mice selectively eliminates pathogenic B220-expressing T cells in vivo [13, 14].

Normal effector T cells entering apoptosis after repeated activation by their antigen express the tyrosine phosphatase B220 on their surface [15, 16, 42], suggesting a role for B220 induction in the transition from activation to apoptosis. On the other hand, B220 is the isoform of CD45 predominantly expressed on pathogenic DN T cells from patients and mice with a deficiency in the death receptor Fas, or its ligand FasL. FasL-deficient mice (*gld* mutation) with only one functional CD45 allele (*gld*/*gld, CD45+/-*) display a strong reduction in the pathogenic DN T-cell population [43], suggesting that CD45 is an important regulator of T-cell apoptosis or a survival factor for T cells. Whether and how B220 expression on T cells regulates signaling for death or survival remains unknown. The induction of B220 and FasL (in EL-4 cells) as well as the activation of initiator caspase-8 in As_2_O_3_-treated leukemic T-cell lines were reminiscent of apoptosis in normal effector T cells triggered by repeated antigenic stimulation. Therefore, leukemic T-cell lines were activated with calcium ionophore A23187, a well-known trigger for T-cell activation and death. Importantly, we found that As_2_O_3_ and calcium ionophore A23187 have opposite efficiencies on the T-cell lines, evidenced by the induction of B220, activation marker CD69 and membrane-bound HSP70 expression, and cell death (data summarized in Table 1). While both B220 expression and cell death were massively induced in EL-4 cells after treatment with As_2_O_3_, they were only slightly induced after treatment with calcium ionophore A23187. The complete reverse situation was observed in HPB-ALL cells, indicating that A23187 and As_2_O_3_ had opposite effects on B220 expression and cell death on the same leukemic T-cell panel. Moreover, treatment with A23187, but not As_2_O_3_, induced the activation marker CD69 on T-cell lines before B220 expression and cell death, indicating that A23187, but not As_2_O_3_, kills the T-cell lines by an activation-induced cell death mechanism.

HSP70 is overexpressed in various cancer cells [44]. HSP70 inhibits apoptosis by modulating multiple events within apoptotic pathways, which might promote cancer development [45, 46]. However, a tumor-specific plasma membrane form of HSP70 has been described [44, 47], which facilitates tumor rejection by the immune system [27, 47–49]. In the present study, we found a strong upregulation of membrane-bound HSP70 by As_2_O_3_ treatment, but not by the calcium ionophore A23187. This upregulation of HSP70 by As_2_O_3_ strictly paralleled the induction of B220 on EL-4, BW5147, Jurkat and HPB-ALL T-cell lines (Table 1). Consequently, As_2_O_3_-sensitive EL-4 cells expressed both high levels of HSP70 and B220, whereas low expression levels of HSP70 and B220 were found on As_2_O_3_-resistant HPB-ALL cells. Likewise, constitutive expression of B220 on As_2_O_3_-resistant L1210 cells was associated with low expression levels of membrane-bound HSP70. In vivo, the direct cytotoxic effects of As_2_O_3_ could be amplified by the upregulation of membrane-bound HSP70 on tumor cells, which might facilitate tumor immune rejection.

CD45 is known to positively regulate antigen-receptor signaling during activation of normal T and B cells via dephosphorylation of src kinases, and to negatively regulate cytokine receptor signaling via dephosphorylation of JAK kinases [17, 18]. In this study, we show that the modulation of B220 cell surface expression plays an important role in determining the sensitivity of leukemic T cells to As_2_O_3_ and calcium ionophore A23187 cytotoxicity. In addition, we found that As_2_O_3_ treatment represses nuclear translocation of NF-κB p50 in a dose dependent manner. Moreover nuclear translocation of NF-κB p50 was increased in CD45-deficient Jurkat T cell line (clone J45.01) after treatment with As_2_O_3_, suggesting a link between B220 and NF-κB signaling pathways.

In conclusion, on a panel of mouse and human leukemic T-cell lines, we have presented evidence for a tight correlation between the induction of B220 membrane expression and their sensitivity to cell death induced by As_2_O_3_ or A23187. Our data strongly support the hypothesis that B220 plays a checkpoint role in death pathways. This could provide additional tools to potentiate As_2_O_3_ therapy against leukemic T cells.

## Conclusions

In contrast to As_2_O_3_-treated APL cells, GSH content and O_2_^-^ production do not play a significant role in As_2_O_3_ sensitivity of leukemic T cells, suggesting the existence of additional factors determining the sensitivity of T cells to As_2_O_3_ cytotoxicity. The B220 isoform of transmembrane tyrosine phosphatase CD45 may be one such factor. Indeed, we show that As_2_O_3_ treatment induces B220 plasma membrane expression and cell death in leukemic T-cell lines in a dose and time dependent manner. The levels of B220 induction on the T-cell lines strictly correlate with both the extent and form of cell death. Leukemic T cells died by an apoptotic form of cell death when high levels of B220 membrane expression were achieved with low doses of As_2_O_3_. Taken together, our data suggest that transmembrane tyrosine phosphatase B220 plays a checkpoint role in apoptotic pathways since its expression is markedly and rapidly induced on the surface of T cells undergoing apoptosis.

## Materials and methods

### Reagents

Arsenic trioxide (As_2_O_3_), phorbol 12-myristate 13-acetate (PMA) and 4′,6-diamidino-2-phenylindole (DAPI) were purchased from Sigma-Aldrich (St. Louis, MO), and Calcium ionophore A23187 was from Calbiochem (EMD Biosciences Inc, San Diego, CA). As_2_O_3_ was dissolved in 1 M NaOH, and stored as a 330 mM stock solution, which was further diluted to 5 mM with phosphate-buffered saline (PBS).

### Cell culture and cell treatment

Leukemic cell lines used in this study included mouse T-cell lines EL-4, BW5147 and L1210, human T-cell lines HPB-ALL, Jurkat (clone E6-1) and CD45 deficient variant of the E6-1 clone of Jurkat (clone J45*.*01) (European Collection of Cell Cultures), and human acute promyelocytic leukemia cell line NB4. Leukemic T cell lines (EL-4, BW5147, L1210, Jurkat (clone E6-1), HPB-ALL), and APL derived cell line NB4 were kindly provided by Dr Colette Kanellopoulos-Langevin (Centre for Inflammation Research, INSERM, Hôpital Bichat, Paris, France) and Dr Jacqueline Robert-Lézénès (Inserm U940, Hôpital Saint-Louis, Paris, France), respectively. All cells were grown in RPMI 1640 containing Glutamax (Invitrogen, Cergy Pontoise, France) and supplemented with 10% heat-inactivated fetal calf serum, 50 U/ml penicillin and 50 μg/ml streptomycin at 37°C in a humidified 5% CO_2_ atmosphere. This culture medium will be referred to as complete medium. To avoid possible effects of cell density on cell growth and survival, cells were maintained at less than 5 × 10^5^ cells/ml with daily adjusting cell density through the addition of fresh medium. Cell viability was estimated by the 4% Trypan-blue dye exclusion assay.

Leukemic T cells were seeded in 12-well plates at a density of 1 × 10^5^ cells /ml and incubated in complete medium alone or in the presence of different concentrations of As_2_O_3_ or calcium ionophore A23187 at 37°C for 12, 24 and 48 h depending on the experiment.

### Flow cytometry and imaging flow cytometry

The expression levels of cell surface markers on untreated and As_2_O_3_- or A23187-treated leukemic T-cell lines was analyzed by flow cytometry using either fluorescein isothiocyanate (FITC)-, phycoerythrin (PE)-, allophycocyanin (APC)- or biotin-conjugated monoclonal antibodies (mAb): rat anti-mouse Thy-1.2/CD90.2 (clone 53–2.1), anti-CD45 (clone 2D1), anti-B220/CD45R (clone RA3-6B2), anti-mouse and human CD69 (clone H1.2 F3 and FN50), anti-mouse and human Fas (clone Jo2 and DX2), anti-mouse and human FasL (clone MFL3 and NOK-1) and anti-HSP70 (clone SMC-103A) (all from eBioscience, CliniSciences, Montrouge, France), and rat IgG2a, mouse IgG, mouse IgG1, and Armenian hamster IgG1 as the isotype control (eBiosciences). Use of mAb to mouse and human Fcγ receptor (PharMingen, BD Bioscience, San Jose, CA) avoided non-specific antibody binding.

The subcellular localization of B220/CD45R molecules was determined by imaging flow cytometry, following the protocol supplied by the manufacturer (Amnis Corp., Seattle, WA). Briefly, cells (1 × 10^6^) stained with PE-conjugated anti-B220/CD45R mAbs and DAPI were run on an ImageStream apparatus (ImagoSeine, Institut Jacques Monod, CNRS-Université Paris Diderot, France). At least 10,000 images were collected per sample at 40× or 60× magnification, and analyzed using IDEAS image-analysis software (Amnis Corp.).

### Cell proliferation, cell death and caspase activation assay

Total cell numbers in untreated and As_2_O_3_-treated groups were determined by flow cytometry by acquiring events for a fixed time period of 1 min. As_2_O_3_- and Calcium ionophore-induced cell death was analyzed by propidium iodide (PI) (Invitrogen) staining, and flow cytometry. Among PI^+^ cells, to discriminate between apoptotic and necrotic cells, the cells were stained using either FITC-conjugated Annexin V (PharMingen) or CaspaTag Caspase 8 or Caspase 9 In situ Assay Kit, Fluorescein according to the manufacturer's instructions (Chemicon, Temecula, CA). Annexin V staining and the levels of active caspase-8 and caspase-9 were measured by flow cytometry.

### Analysis of reduced glutathione content and O_2_^-^ production

GSH content and O_2_^-^ production in T-cell lines treated or not with As_2_O_3_ were measured by flow cytometry using 100 nM CellTracker probe CMFDA and 5 μM DHE probes, respectively, following the manufacturer’s instructions (Molecular Probes, Eugene, OR).

### B220/CD45R mRNA quantification by RT-PCR

Total RNA was extracted from 5 × 10^6^ leukemic T cells treated or not with As_2_O_3_ using the RNeasy Plus Mini kit (Qiagen, Courtaboeuf, France) following the manufacturer’s instructions and was used to generate cDNA utilizing oligo(dT) primer and SuperScript II Reverse Transcriptase (Invitrogen). PCR were conducted for 25 cycles with the following primer pairs: B220/CD45R forward primer (5'-CAC ATA TCA TCC AGG TGT GTT ATC C-3') and reverse primer (5'-GTC CTC TCC CCT GGC ACA CCT G-3'); β-actin forward primer (5'-ATC GTG GGC CGC CCT AGG CAC-3') and reverse primer (5'-TGG CCT TAG GGT TCA GAG GGG C-3'). Semi-quantitative determination (ImageJ densitometric analysis software program) of B220 cDNA, present in each of the various samples, was normalized with respect to the concentration of internal control cDNA (β-actin) detected in the same sample, and B220/ β-actin cDNA ratios were calculated.

### *Quantitative measurement of NF*-*κB*activation

Nuclear extracts were prepared from 8 × 10^6^ leukemic T cells treated or not with As_2_O_3_. The protein from nuclear extracts was quantified by the Bradford method (Bio-Rad, France). An equal amount of nuclear extract (5 μg) was assayed for NF-κB p50 activity using a TransAm NFκB p50 Transcription Factor Assay Kit according to the manufacturer’s recommendations (ActiveMotif, Rixensart, Belgium).

### Statistical analyses

Data are reported as fluorescence means ± SE. Significant differences between sample means were determined using the Student *t* test. Statistical significance was accepted at *P* ≤0.05.

## Electronic supplementary material

Additional file 1: Figure S1: (A) Cell morphology of As_2_O_3_-treated T-cell lines. APL-derived NB4 cells as well as EL-4, BW5147, L1210, Jurkat, CD45-deficient Jurkat variant (J45.01) and HPB-ALL T-cell lines were treated for 24 h with As_2_O_3_ in doses ranging from 1 to 20 μM. Cells were then analyzed by flow cytometry with respect to size (FSC) and granulosity (SSC). Regions R1 and R2 identified on a FSC vs. SSC dot plot encompassed cells with FSC^int/low^SSC^high^ and FSC^high^SSC^low^, respectively. At least 20,000 events were analyzed for each sample. FSC vs. SSC dot plots on murine EL-4 cells and human Jurkat cells are representative of more than 10 independent experiments. (B) Basal level of CD45 plasma membrane expression. Jurkat (■) and CD45-deficient Jurkat variant (J45.01) (□) cells were stained with PE-conjugated anti-CD45 mAb (clone 2D1) or PE-conjugated rat IgG2a isotype control, and then analyzed by flow cytometry. (C) B220 mRNA expression in As_2_O_3_-treated T-cell lines. RT-PCR analysis were performed to assess the levels of B220 mRNA in EL-4 and BW5147 T cells cultured in the presence or absence of 1, 2 and 4 μM As_2_O_3_ for 3, 6 and 9 h. Results are representative of two other experiments. (TIFF 2 MB)

Additional file 2: Figure S2: (A) Constitutive B220/CD45R cell surface expression on CD90^+^ L1210 T cells. Cells were labeled with APC-conjugated anti-CD90 and PE-conjugated anti-B220/CD45R mAbs, or fluorescent isotype control, and then analyzed by flow cytometry. (B) B220 expression on As_2_O_3_-treated cells. L1210 T cells were treated without or with As_2_O_3_ for 24 h in doses ranging from 1 to 20 μM. L1210 cells were then stained with PE-conjugated anti-B220/CD45R mAb or PE-conjugated rat IgG2a isotype control, and further analyzed by flow cytometry with respect to size (FSC) versus granulosity (SSC) and B220 expression. FSC vs. SSC dot plots were used to define gates R1 and R2 with FSC^int/low^SSC^high^ and FSC^high^SSC^low^, respectively. B220 histograms were then gated in R1 and R2 to determine the percentages of cells expressing B220 (n =10 independent experiments). At least 20,000 events were analyzed for each sample. (C) HSP70 induction on As_2_O_3_-treated cells. L1210 T cells stained with anti-B220 and anti-HSP70 antibodies were analyzed by flow cytometry as described in Figure 3B. (D) FasL induction on Ca^2+^ ionophore treated cells. Histograms obtained with PE-conjugated Armenian hamster (clone MFL3) anti-FasL mAb (open histogram) are overlaid on histograms obtained with PE-conjugated Armenian hamster isotype control (shaded histogram) (n = 3 independent experiments). At least 20,000 events were analyzed for each sample. (TIFF 2 MB)

Additional file 3: Figure S3: Duration of B220/CD45R membrane expression upon As_2_O_3_ treatment. EL-4 and Jurkat T cells were cultured in the absence or in the presence of 1, 2, 4 or 8 μM As_2_O_3_ for 24 h. Then, cells were extensively washed with PBS to eliminate all traces of As_2_O_3_, and cultured for 9 additional days. Expression of B220 was measured by flow cytometry at the time of As_2_O_3_ removal (referred to as day 0) and 1 to 9 days after As_2_O_3_ was removed. At least 20,000 events were analyzed for each sample. Dot plots of FSC vs. SSC on 8 μM As_2_O_3_-treated EL-4 and Jurkat cells are representative of more than 3 independent experiments. Graphs report the percentages of B220^+^ EL-4 or B220^+^ Jurkat cells at the indicated time-points and concentrations of As_2_O_3_, with the same isotype control labelling as in Figure 3. (TIFF 2 MB)

## Abbreviations

APL: Acute promyelocytic leukemia
AQP9: Aquaglyceroporin-9
As_2_O_3_: Arsenic trioxide
DN: Double negative
FasL: Fas ligand
FSC: Forward scatter
EC_50_: Half maximal effective concentration
IC_50_: Half maximal inhibitory concentration
HSP70: Heat shock protein-70
mAb: Monoclonal antibody
NF-κB: Nuclear factor-kappa B
PI: Propidium iodide
ROS: Reactive oxygen species
GSH: Reduced glutathione
SSC: Side scatter
O_2_^-^: Superoxide anion radical
MFI: Mean fluorescence intensity.
