# Supplementary figures and images for: Sensitivity of leukemic T-cell lines to arsenic trioxide cytotoxicity is dependent on the induction of phosphatase B220/CD45R expression at the cell surface
Source: Mol Cancer. 2014 Nov 19;13:251. doi: 10.1186/1476-4598-13-251 (PMC4252024; doi:10.1186/1476-4598-13-251)

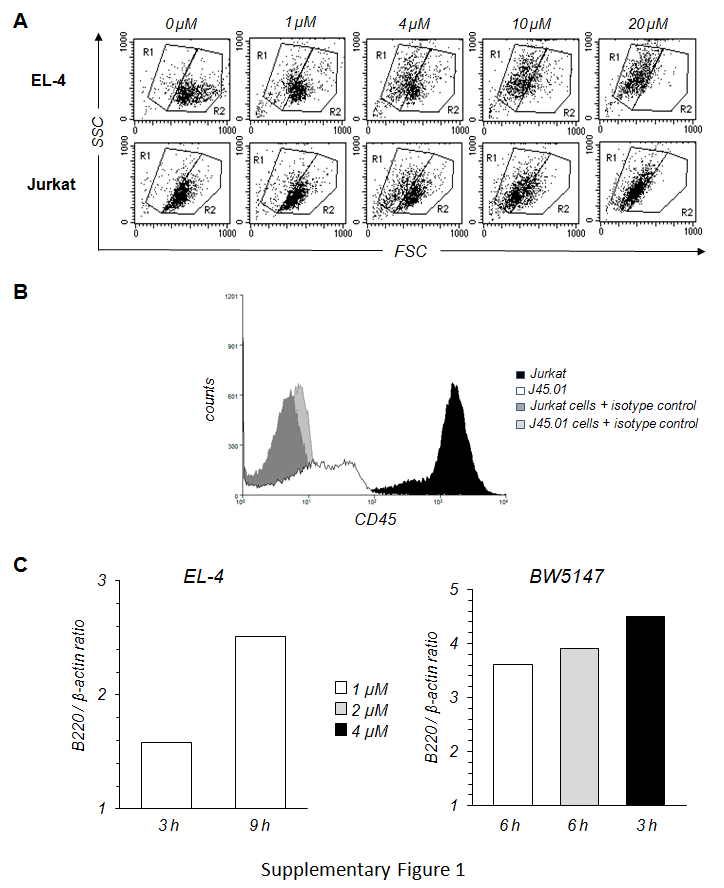

Supplement: Supplementary file 1 — Additional file 1: Figure S1: (A) Cell morphology of As2O3-treated T-cell lines. APL-derived NB4 cells as well as EL-4, BW5147, L1210, Jurkat, CD45-deficient Jurkat variant (J45.01) and HPB-ALL T-cell lines were treated for 24 h with As2O3 in doses ranging from 1 to 20 μM. Cells were then analyzed by flow cytometry with respect to size (FSC) and granulosity (SSC). Regions R1 and R2 identified on a FSC vs. SSC dot plot encompassed cells with FSCint/lowSSChigh and FSChighSSClow, respectively. At least 20,000 events were analyzed for each sample. FSC vs. SSC dot plots on murine EL-4 cells and human Jurkat cells are representative of more than 10 independent experiments. (B) Basal level of CD45 plasma membrane expression. Jurkat (■) and CD45-deficient Jurkat variant (J45.01) (□) cells were stained with PE-conjugated anti-CD45 mAb (clone 2D1) or PE-conjugated rat IgG2a isotype control, and then analyzed by flow cytometry. (C) B220 mRNA expression in As2O3-treated T-cell lines. RT-PCR analysis were performed to assess the levels of B220 mRNA in EL-4 and BW5147 T cells cultured in the presence or absence of 1, 2 and 4 μM As2O3 for 3, 6 and 9 h. Results are representative of two other experiments. (TIFF 2 MB) [file 12943_2014_1451_MOESM1_ESM.tiff]

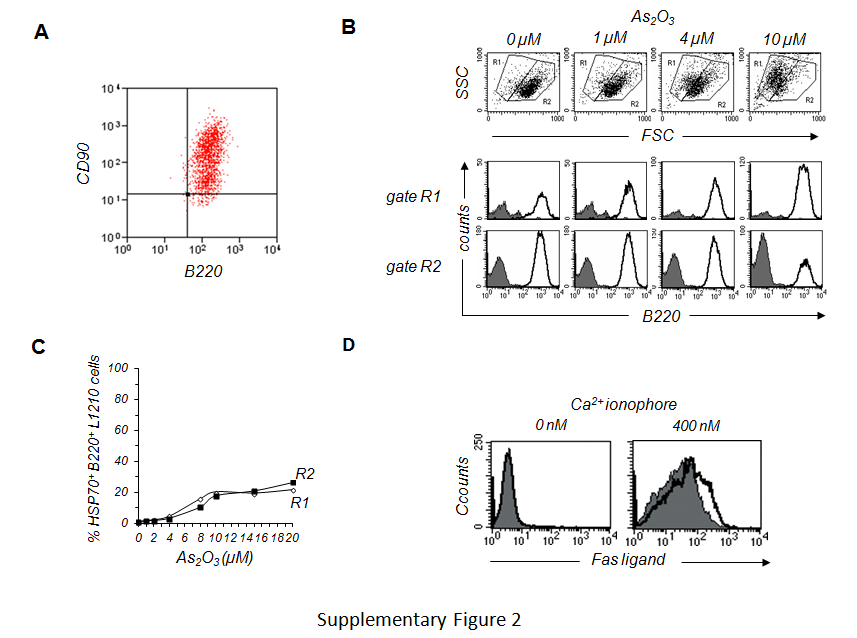

Supplement: Supplementary file 2 — Additional file 2: Figure S2: (A) Constitutive B220/CD45R cell surface expression on CD90+ L1210 T cells. Cells were labeled with APC-conjugated anti-CD90 and PE-conjugated anti-B220/CD45R mAbs, or fluorescent isotype control, and then analyzed by flow cytometry. (B) B220 expression on As2O3-treated cells. L1210 T cells were treated without or with As2O3 for 24 h in doses ranging from 1 to 20 μM. L1210 cells were then stained with PE-conjugated anti-B220/CD45R mAb or PE-conjugated rat IgG2a isotype control, and further analyzed by flow cytometry with respect to size (FSC) versus granulosity (SSC) and B220 expression. FSC vs. SSC dot plots were used to define gates R1 and R2 with FSCint/lowSSChigh and FSChighSSClow, respectively. B220 histograms were then gated in R1 and R2 to determine the percentages of cells expressing B220 (n =10 independent experiments). At least 20,000 events were analyzed for each sample. (C) HSP70 induction on As2O3-treated cells. L1210 T cells stained with anti-B220 and anti-HSP70 antibodies were analyzed by flow cytometry as described in Figure 3B. (D) FasL induction on Ca2+ ionophore treated cells. Histograms obtained with PE-conjugated Armenian hamster (clone MFL3) anti-FasL mAb (open histogram) are overlaid on histograms obtained with PE-conjugated Armenian hamster isotype control (shaded histogram) (n = 3 independent experiments). At least 20,000 events were analyzed for each sample. (TIFF 2 MB) [file 12943_2014_1451_MOESM2_ESM.tiff]

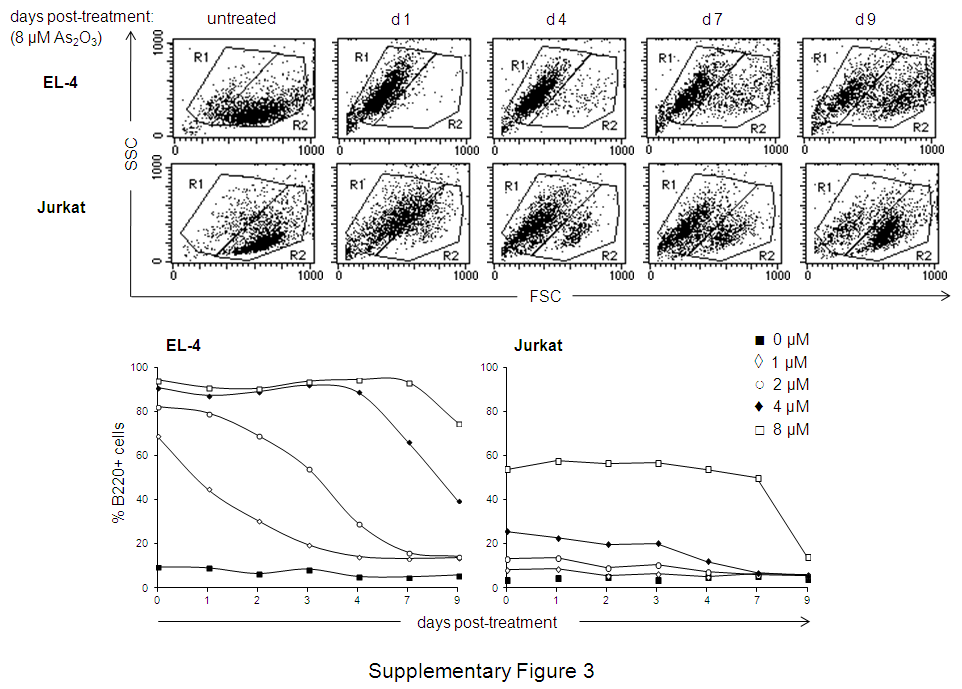

Supplement: Supplementary file 3 — Additional file 3: Figure S3: Duration of B220/CD45R membrane expression upon As2O3 treatment. EL-4 and Jurkat T cells were cultured in the absence or in the presence of 1, 2, 4 or 8 μM As2O3 for 24 h. Then, cells were extensively washed with PBS to eliminate all traces of As2O3, and cultured for 9 additional days. Expression of B220 was measured by flow cytometry at the time of As2O3 removal (referred to as day 0) and 1 to 9 days after As2O3 was removed. At least 20,000 events were analyzed for each sample. Dot plots of FSC vs. SSC on 8 μM As2O3-treated EL-4 and Jurkat cells are representative of more than 3 independent experiments. Graphs report the percentages of B220+ EL-4 or B220+ Jurkat cells at the indicated time-points and concentrations of As2O3, with the same isotype control labelling as in Figure 3. (TIFF 2 MB) [file 12943_2014_1451_MOESM3_ESM.tiff]
